# The cytidine deaminase under-representation reporter (CDUR) as a tool to study evolution of sequences under deaminase mutational pressure

**DOI:** 10.1186/s12859-018-2161-y

**Published:** 2018-05-02

**Authors:** Maxwell Shapiro, Stephen Meier, Thomas MacCarthy

**Affiliations:** 1 Department of Applied Mathematics and Statistics, Stony Brook University, 100 Nicolls Road, Stony Brook, NY, USA; 2 Laufer Center for Physical and Quantitative Biology, Stony Brook University, 100 Nicolls Road, Stony Brook, NY, USA

**Keywords:** AID, APOBEC, Deaminase, Mutation motifs, Virus

## Abstract

**Background:**

Activation induced deaminase (AID) and apolipoprotein B mRNA editing enzyme, catalytic polypeptide-like 3 (APOBEC3) are deaminases that mutate C to U on single-stranded DNA (ssDNA). AID is expressed primarily in germinal center B-cells, where it facilitates affinity maturation and class-switch recombination. APOBEC3 are a family of anti-viral proteins that act as part of the intrinsic immune response. In both cases, there are particular sequence motifs, also known as “mutation motifs”, to which these deaminases prefer to bind and mutate.

**Results:**

We present a program, the cytidine deaminase under-representation reporter (CDUR) designed to statistically determine whether a given sequence has an under/over-representation of these mutation motifs. CDUR shows consitency with other studies of mutation motifs, as we show by analyzing sequences from the adeno-associated virus 2 (AAV2) and human papillomavirus (HPV).

**Conclusion:**

Using various shuffling mechanisms to generate different null model distributions, we can tailor CDUR to correct for metrics such as GC-content, dinucleotide frequency, and codon bias.

## Background

In both innate and adaptive immunity, vertebrates utilize cytidine deaminase enzymes as part of the immune response against viral infections. In the innate immune system, the family of apolipoprotein B mRNA editing enzyme, catalytic polypeptide-like (APOBEC) proteins, primarily belonging to the sub-family of APOBEC3 proteins, act on single-stranded DNA (ssDNA) by mutating C to U, resulting in C >T transitions following replication [1]. In the adaptive immune system, the cytidine deaminase AID (activation induced deaminase) similarly mutates the antibody (immunoglobulin) genes in B-cells during the germinal center reaction to generate antigen-specific antibodies. Within the germinal center, B-cells also proliferate rapidly and are selected based on the affinity of their antibody receptor for antigen, thus, AID, the ancestral gene within the APOBEC family [2, 3], enables antibody affinity maturation. Both AID and APOBEC3 proteins share a structure of a central *β* sheet flanked by 4-5 *β* sheets and 6-7 *α* helices. The catalytic pocket of these proteins contains a zinc ion that facilitates binding to negatively charged nucleotides. The catalytic action of AID, as determined biochemically, suggests that AID binds to ssDNA with high affinity, but has very low catalytic rate. Consistent with this, molecular dynamics simulations suggest that the binding pocket of AID is occluded 75% of the time, which presumably protects against excessively high levels of deamination [4].

In mammals, the APOBEC3 sub-family has duplicated and diverged such that in primates, including human, there are seven different APOBEC3 genes: APOBEC3A, 3B, 3C, 3DE, 3F, 3G, and 3H [5, 6]. Associated with each of these genes there is a motif-specific “mutation motif” (MM) which the enzyme preferentially mutates (in the B-cell immunology literature these are often referred to as “hotspots”). Table 1 shows the preferred mutation motifs for the seven human APOBEC3 proteins and AID. It is worth mentioning that, in the case of AID, there are mutational “coldspots” which are sites at which AID tends to avoid during hypermutation. These “coldspots” have a mutation motif of SYC (S=G/C, Y=T/C) [7]. Though APOBECs were identified to be utilized as mostly anti-viral factors, some cancer-causing mutations have also been attributed to APOBEC3 proteins. For example, in the case of breast cancer, there is evidence that APOBEC3 enzymes are a significant driver in certain cancer mutations. Further, it has been shown that some cancers have an abundance of closely-spaced, clustered mutations, which are termed “kataegis”. Kataegis mutations have been observed in tumor genes where it is assumed that regions of ssDNA are prone to become exposed. Kataegis mutations appear to be enriched at TCW (W= A or T) motifs where APOBEC3B and APOBEC3A may deaminate processively. The clusters tend to consist entirely of mutations on one DNA strand within these TCW motifs, consistent with the TC mutation motifs of APOBEC3B and APOBEC3A [8–13].

**Table 1:** APOBEC3/aid mutation motifs

APOBEC3/AID	Mutation motif
AID	WRC [7]
APOBEC3A	TC [27]
APOBEC3B	TC [28]
APOBEC3C	TTC [29]
APOBEC3D	TC [30]
APOBEC3F	TTC [31]
APOBEC3G	CCC [31–33]
APOBEC3H	TC [30]
Murine APOBEC3	TYC [34, 35]

Given a sequence, one may want to investigate the consequences on the sequence of evolving under the mutational pressure of cytidine deaminases by analyzing the sequence in terms of AID/APOBEC mutation motifs. For example, when studying Epstein-Barr virus (EBV), one may find that some of its coding sequences have evolved to limit the number of AID mutation motifs (defined by WRC), since EBV establishes latency and reactivates in B-cells, potentially exposing the EBV genome to AID [14, 15]. In addition, it would be important to determine if there is evidence that the genome has an under-representation in the total number of mutation motifs, or in those mutation motifs that may cause nonsynonymous mutations [15]. This can be helpful in determining cancer treatments in which APOBECs are targeted for oncotherapy [16]. We can also determine over-representation, which is defined to be an excess of certain type of mutation motif, beyond what would be expected given a null model. Conditional biases may also exist between different mutation motifs when searching for over-/under-representation, especially if the definitions overlap. For instance, if EBV were to gain an over-representation of AID coldspots in certain genes, that may also cause an enrichment of APOBEC3G mutation motifs since CCC is a subset of the AID coldspot motif SYC. Methods used to study under-/over-representation are discussed in [15, 17]. In these previous studies, the authors used coding sequences to generate null distributions of mutation motifs that could then be compared to the input sequence in order to determine mutation motif under-/over-representation. These previous methods corrected primarily for GC content but did not account for the relevance of dinucleotide frequency, codon bias, or codon pair bias. Assuming these additional features may be biologically relevant, it would be useful to incorporate these into null model generation when quantifying mutation motif over-/under-representation.

We have developed a program, the Cytidine Deaminase Underrepresentation Reporter (CDUR), that analyzes gene coding sequences to determine if the sequence has a statistical under-representation (or over-representation) for cytidine deaminase mutation motifs. The statistical method involves generating a null distribution for the number of mutation motifs within the sequence being analyzed (the subject), by repeatedly shuffling the sequence so as to preserve the amino acid sequence. The subject sequence is then compared to the null distribution to generate a P value. In addition to the number of cytidine deaminase mutation motifs, we also consider statistics for the number of nonsynonymous mutations occurring at those mutation motifs, as well as the ratio of nonsynonymous mutations to mutation motifs. We then use this program to analyze the Rep-68 protein in the adeno-associated virus 2 (AAV2) and the human papillomavirus (HPV) E6 proteins, which were chosen to compare our results to a previous study [15] that used a simpler model.

## Methods

The Cytidine Deaminase Underrepresentation Reporter (CDUR) is composed of two software modules: a shuffling algorithm, and a statistical reporter. The shuffling algorithm generates the null distribution, i.e., given our subject coding sequence, we generate biologically feasible sequences subject to particular constraints. In defining our null model, we assume that other nucleotide sequences that preserve the amino-acid sequence, might have been equally possible through the course of evolution. Thus, we consider how our observed sequence (the subject) compares to these other biologically feasible sequences (the null model). After we obtain our null distribution via the shuffling, we then perform the statistical analysis that yields the metrics described below.

### Coding sequence shuffling

The shuffling methods we discuss in this paper were proposed previously [18, 19]. We next describe briefly the features of each of the three shuffling methods that are available within CDUR to generate null distributions for mutation motif counts. Note that all three methods are applied to a coding sequence (hereafter referred to as the subject sequence) and maintain the integrity of the underlying amino acid sequence by choosing new, synonymous codons based on the criteria described below.

#### gc3

**Fig. 1:**
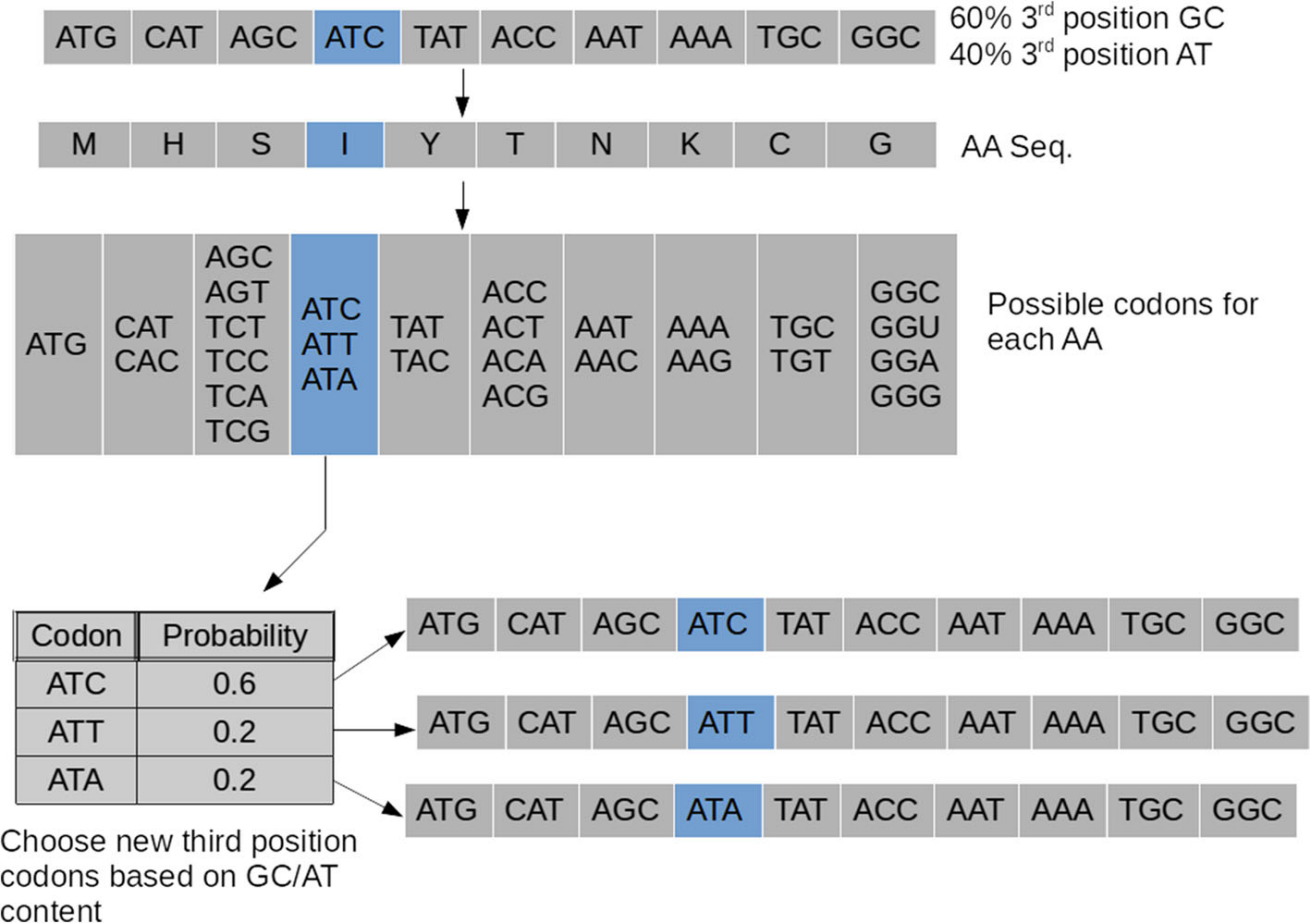
gc3 shuffle method. The choice of codons in the 4th nucleotide in the sequence (Ile) was determined by the probabilities as follows: since there is an overall GC content of 60% at the 3rd position of the codons in the subject sequence, the ATC codon will be chosen with 0.6 probability. Since the AT content is then 0.4, the other two codons ATT and ATA are chosen randomly with equal probability, conditional on the 40% AT content. Note that the shuffling occurs iteratively throughout sequence, not just one codon at a time

The gc3 method first considers the GC-content of the subject sequence in the third codon position. As discussed in a previous study, the overall GC-content of a sequence is related to mutation motif over- or under-representation [15]. This shuffle method changes codons while correcting for GC-content as follows: for each codon, the first two positions are always preserved. The third position is chosen randomly subject to the GC-content of the third position codon for all codons in the entire sequence. Thus, in the example of Fig. 1, the sequence contains 10 codons, of which 6 (60%) have G or C at the 3rd position. If we fix positions 1 and 2 of any codon, synonymous changes at the 3rd position must (based on the genetic code) fall into one of the following categories: R (A/G), Y (T/C), H (A/C/T), or N (any nucleotide). Note that six-codon amino acids such as Serine are considered as a combination of a two- and a four-codon amino acid. For the highlighted (I) codon in Fig. 1, this would be H (A/C/T). The gc3 method chooses the third position synonymously from R, Y, H, or N, distributing the probabilities based on the GC-content of the third codon position in the entire sequence (here, 60%), as shown in Fig. 1. This method corrects for the GC-content of the sequence, albeit not necessarily conserving it exactly due to sampling effects. Furthermore, this method does not necessarily conserve other amino acid sequence attributes such as codon bias, codon pair bias and dinucleotide bias (Fig. 1).

#### n3

**Fig. 2:**
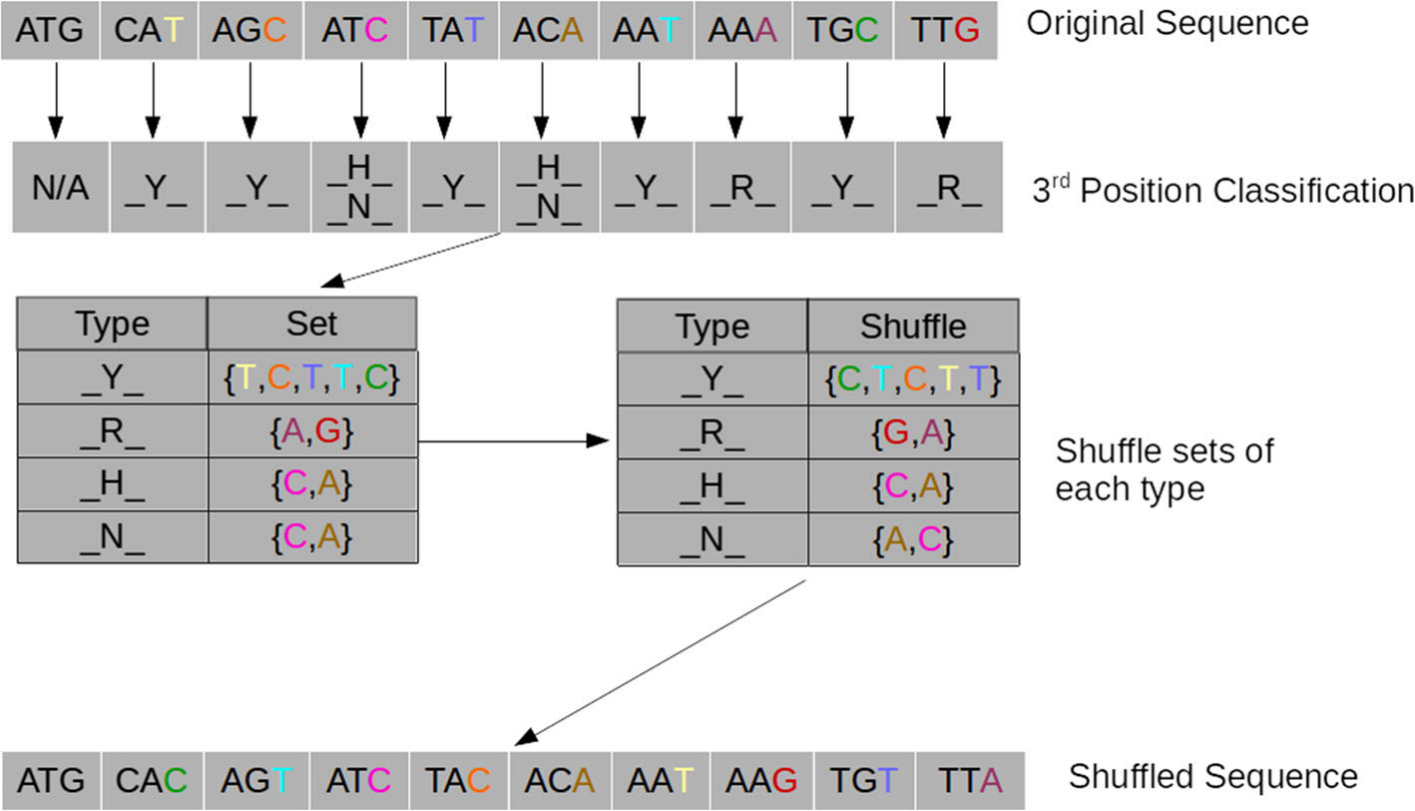
n3 shuffle method. Third codon positions are first categorized into whether they belong to Y, R, H, or N nucleotides, then assigned to the corresponding set (Type vs Set table). Then nucleotides within each set are shuffled (Type vs Shuffle table) to produce the shuffled sequence. Certain nucleotides can belong to two sets (here two nucleotides belong to both H and N) and are shuffled twice

Similar to gc3, the n3 shuffle method also considers third position codons, and again indexes third position codons into sets for R, Y, H, and N nucleotides. However, instead of choosing third positions based on GC-content, this method starts by recording the third position nucleotide for each type to construct a set of these nucleotides (see Type vs Set table in Fig. 2), which are then shuffled and randomly assigned without replacement among the codons of the same type (R, Y, H or N), as shown in Fig. 2. This does not change the number of each nucleotide in the sequence and specifically, leaves GC-content unchanged. However, it does not necessarily maintain codon bias or dinucleotide frequencies [19].

#### dn23

**Fig. 3:**
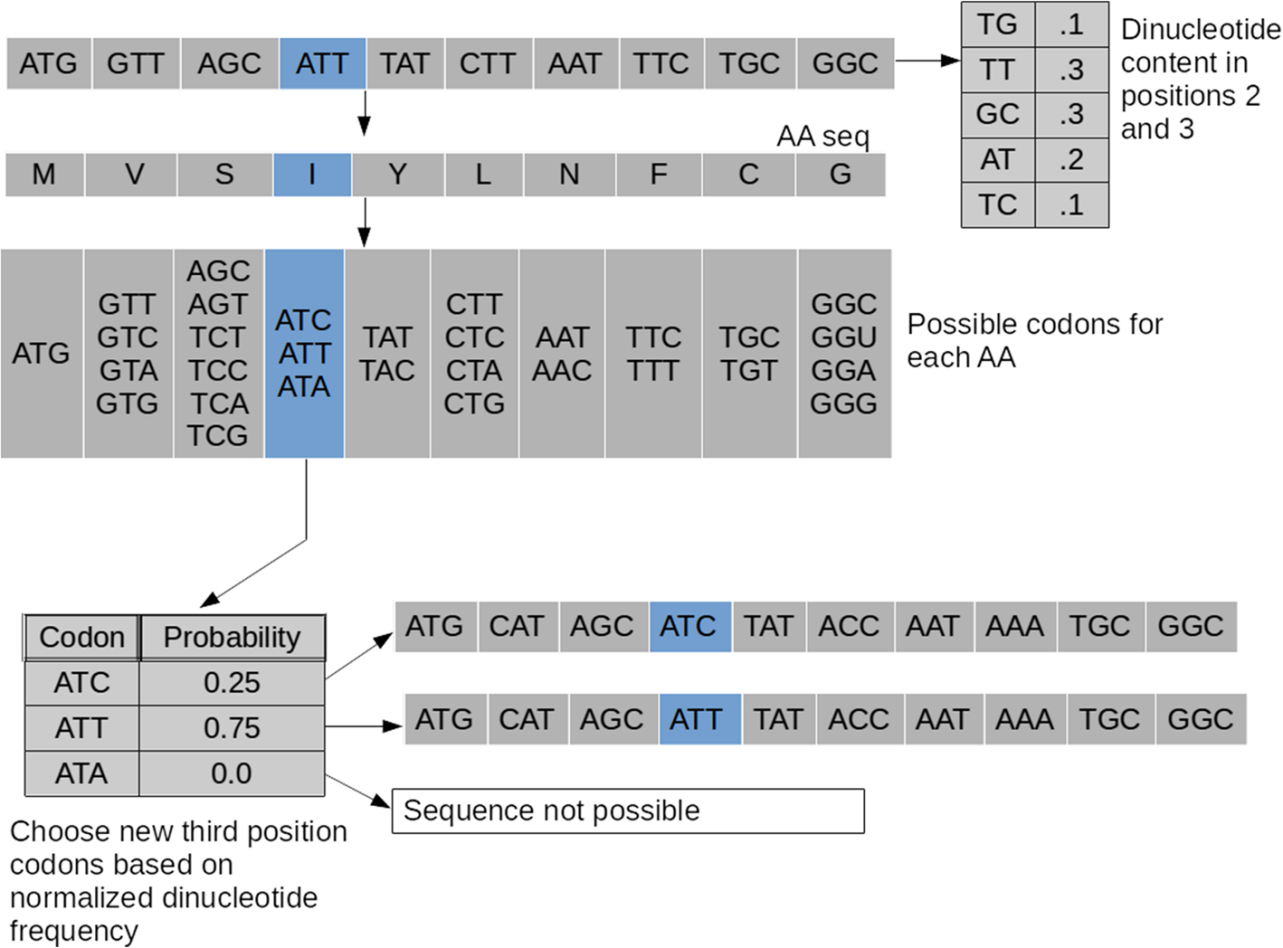
dn23 shuffle method. First the dinucleotide frequency is calculated for the 2nd and 3rd codon positions of the original sequence. Then for each amino acid, codons are chosen based on the appropriately normalized probabilities for the dinucleotides available for that amino acid

The dn23 method is useful for shuffling while largely maintaining the dinucleotide frequency of the subject sequence [19]. The method proceeds by firstly, measuring the dinucleotide frequency of the second and third position codons in the sequence (Fig. 3). Once the dinucleotide frequency has been quantified, the method randomly and synonymously chooses third position codons weighted according to the dinucleotide frequency of the possible options at the second and third positions. Thus, in the highlighted example of Fig. 3, the weight associated with ATT is three times that of ATC because that is the ratio (0.3:0.1) of TT to TC dinucleotides at positions 2 and 3 within the original sequence. This method largely conserves both the dinucleotide frequency and codon bias [19], but it does not necessarily maintain GC-content.

### Cytidine over/under-representation reporter

#### Univariate statistics

For each motif under consideration, and given a subject sequence and shuffle method, we make r new, shuffled sequences (default is *r*=1000). For each shuffled sequence we measure the following: (a) number of mutation motifs, (b) number of replacement, or nonsynonymous, transitions, i.e., given a C to T mutation at the mutation motif, the number of these mutations that are nonsynonymous, and (c) the fraction of replacement transitions, i.e., the number of nonsynonymous C to T mutations divided by the number of mutation motifs, or (b)/(a). The default program considers all possible NC and NNC motifs (N= any nucleotide) as well as WRC (AID mutation motif) and SYC (AID coldspot) motifs. Measurements for these motifs consider both strands. We also quantify the motifs CG (to account for CpG dinucleotides). Also included in the CDUR package is a configuration file that allows the user to choose the motifs and strands to be analyzed. For each measurement, a null distribution is constructed empirically from the r shuffled sequences. From the null distribution, we estimate over- and under- representation by comparing the measurement (e.g. number of mutation motifs) of the subject sequence to the null distribution, together with other statistics, as shown in Table 2. The program produces these as a list of keyword/value pairs. Of note are the statistics belowX, repTr_belowX, and repTrFrac_belowX (where X is the mutation motif motif under consideration, e.g. “belowTTC”). Each of these corresponds respectively to the *P*-value of under-representation for the three calculations described above: (a), (b), and (c). Specifically, this number equals the fraction of sequences in the null distribution with values less than our subject sequence, which is an empirical estimate of the *P*-value. In general, a sequence is considered to be under-represented in a metric, i.e., has fewer observed instances of that metric than expected, if the *P*-value is less than some threshold *q*, typically 0.05. Similarly, we say a sequence is overrepresented if the *P*-value is greater than 1−*q* (e.g. 0.95).

**Table 2:** CDUR metric description

CDUR metric	Description
belowX	% sequences with fewer numbers of MMs than subject for motif X
repTr_below	% sequences with fewer non-synonymous transitions than subject
repTrFrac_belowX	% sequences with fewer nonsynonymous transitions:MMs
corXxY, corRepTrXxy, corRepTrFracXxY	correlation coefficient between MM X and Y
pXcondY, pXcondRepTrY, pXcondRepTrFracY	Bivariate conditional *P*-valueof MM X on Y
expectedX, repTr_expectedX, RepTrFrac_expectedX	Mean MMs, non-syn. transitions, and repTr:MMs in null dist. for X
observedX, repTr_observedX, repTrFrac_observedX	Total MMs, non-syn. transitions, and repTr:MMs in subject for X

#### Bivariate statistics

In addition to calculating the statistics discussed above, our program also calculates two additional bivariate metrics: correlations and conditional *P*-values. For correlations, the pairwise combinations of mutation motifs are considered. The correlation coefficients are calculated for the below, repTr_below, and repTrFrac_below values, and are designated as corXxY, corRepTrXxY, and corRepTrFracXxY respectively for all distinct motifs X and Y (Table 2). These correlations are also used to approximate the joint distribution between any two motifs using the bivariate normal distribution, which in turn is used this to calculate the *P*-values for each of the statistics (below, repTr_below, and repTrFrac_below) for one motif conditional on the observed level of another motif (Table 2). In other words, we estimate the under- or over-representation of a specific motif conditional on the level of another motif that may be a confounding factor. For example, CpG motifs may be selected evolutionarily within a gene as targets for methylation, but this level of CpGs may act as a confounding factor by affecting under- or over-representation of an APOBEC motif, for example, AGC. Therefore, using the normal approximation of the joint distribution, we calculate the conditional distribution for AGC in which all the sequences considered have the same observed CpG metric as our subject. From this conditional distribution, we compute the statistics for the motif of choice.

## Results

### Analysis of AAV2 Rep-68 and HPV E6 proteins

**Fig. 4:**
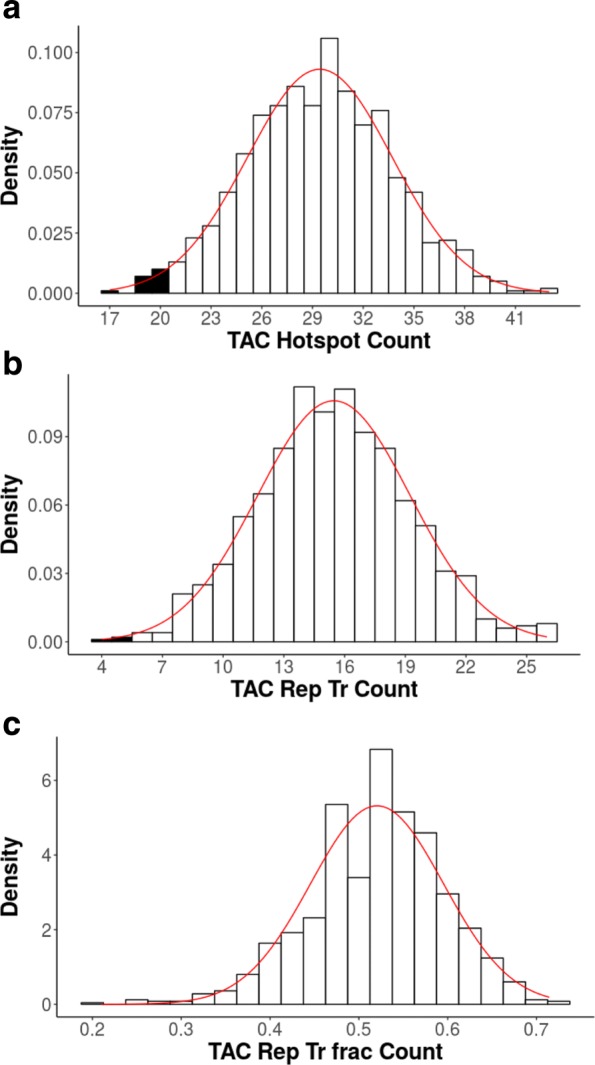
Rep-68 Under-representation. Histograms showing the results of CDUR applied to the AAV2 Rep-68 gene using the gc3 shuffle method. In each graph, the filled black bars represent the “below” portion, that is, the number of shuffled sequences with that given metric below that of the one computed for the input. The red line shows the Normal distribution approximation given the mean and standard deviation (SD) from that calculation. **a** The mutation motif counting graph (belowTAC). The observed number of TAC mutation motifs is 20 (which includes the GTA mutation motifs on the bottom strand). The black bars are all shuffled sequences with fewer observed mutation motifs than our input. **b** The replacement transition graph (RepTr_belowTAC), also shown with the Normal approximation. **c** The replacement transition fraction graph (RepTrFrac_belowTAC). Our sequence had an observed RepTrFrac of 0.25, so the sequences with a lower RepTrFrac are filled in with black (see Table 2 for complete results)

**Fig. 5:**
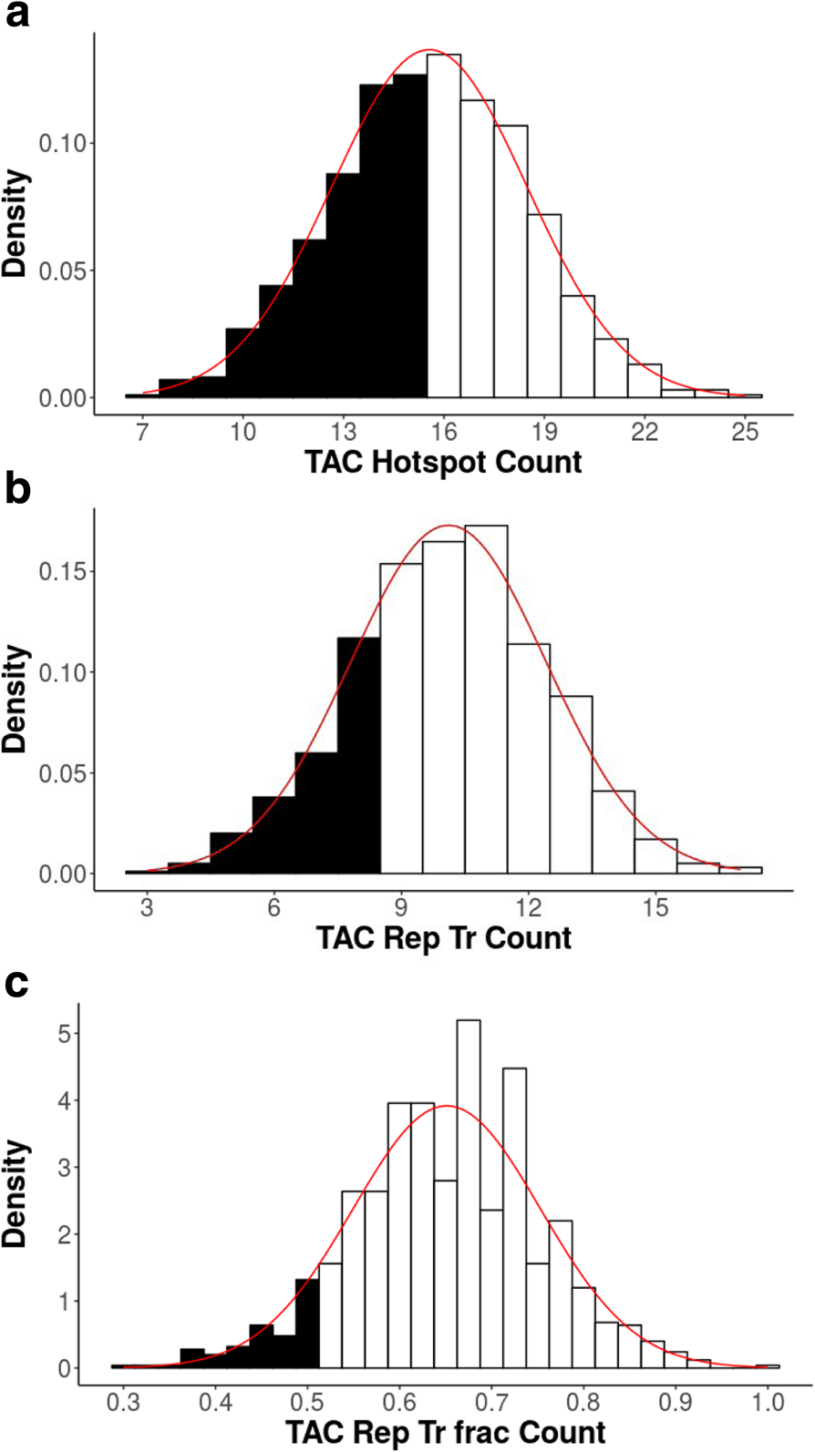
E6 Under-representation. Histograms showing the results of CDUR applied to the HPV E6 gene using the gc3 shuffle method. All subplots (**a**-**c**) and annotations are equivalent to those of Fig. 4, but for this gene. In contrast to Fig. 4 however, rather than under-representation, we see a neutral representation for TAC mutation motifs. (See Table 3 for complete results)

We analyze Adeno-associated virus 2 (AAV2), a member of the Parvovirus family that infects human hosts. This virus is a single-stranded DNA (ssDNA) virus that can appear either as a positive or negative sense virus. Given that the substrate of the AID/APOBEC cytidine deaminases is ssDNA, this virus is a potential target for deamination. The virus contains just two open reading frames (ORFs): Rep and Cap, each of which comes in multiple isoforms. The Rep proteins are involved in DNA replication, whereas Cap are capsid proteins. AAV2 is generally non-pathogenic and is a satellite virus that usually infects those with adenovirus or herpesvirus [20]. Since AAV2 is single stranded, it should be more susceptible to deamination by cytidine deaminases, whose substrate is ssDNA. We analyze the Rep-68 isoform for evidence of underrepresentation for the TAC motif, an AID mutation motif that was identified in a previous study as being potentially susceptible to replacement mutations in this virus based on a simpler shuffling method [15] (Table 3, Fig. 4). As expected, the gc3 shuffle method, the simplest of the CDUR methods, indeed shows the susceptibility of this gene to TAC mutation motifs, consistent with the susceptibility of this gene identified previously. Specifically, we see that the repTrFrac_belowTAC statistic, which is the *P*-value for nonsynonymous mutations to mutation motif ratio (see Methods) is P=0.002 (Table 3). Overall mutation motif under-representation (belowTAC) is also significant (P=0.008). As a negative control for the method we also analyze the Human Papillomavirus (HPV) gene E6 for the same AID mutation motif (Table 3, Fig. 5). Since HPV infects epithelial cells, we do not expect evolution against AID, since AID is only active in proliferating germinal center B-cells. Indeed, we find that the belowTAC values computed for the E6 gene is 0.36, about neutral for representation of TAC mutation motifs. We also see that the repTrFrac_belowTAC value is 0.11, which is not statistically significant.

**Table 3:** CDUR results for Rep-68 and E6 proteins

Metric	AAV2-Rep68	HPV-E6
observedTAC	20.0	15.0
repTr_observedTAC	5.00	8.00
repTrFrac_observedTAC	0.25	.533
expectedTAC	29.4	15.6
repTr_expectedTAC	15.5	10.1
repTrFrac_expectedTAC	.521	.651
expectedSdTAC	4.28	2.92
repTr_expectedSdTAC	3.76	2.30
repTrFrac_expectedSdTAC	.074	.102
belowTAC	.008	0.36
repTr_belowTAC	.001	.124
repTrFrac_belowTAC	.002	.110
corTACxWRC	.364	.277
corRepTrTACxWRC	.282	.053
corRepTrFracTACxWRC	.109	-.272
pTACcondWRC	.086	.182
pTACcondRepTrWRC	.016	.148
pTACcondRepTrFracWRC	.000	.304

## Discussion

Over- and /under-representation of mutation motifs for AID and APOPBEC3 enzymes can give insight into the evolutionary process of an organism. In viruses, we can determine which genes may have been targeted by APOBEC3 enzymes, and how that organism may have evolved to avoid such targeting. Such a virus may evolve to gain an under-representation of the APOBEC3 specific mutation motifs [15]. Though a virus may gain an under-representation by adjusting its GC-content, codon bias, or codon pair bias, the evolutionary pressure from AID/APOBEC3 may be a driving force to gain such an under-representation. As such, CDUR can correct for these co-variates. These methods are also useful in determining over-representation of mutation motifs, which may indicate higher potential for mutation. Relevent to this, signatures of mutational processes in human cancer have been well quantified, and cancer mutations associated with APOBEC mutation motifs can be determined [21]. Models of mutational processes operative in cancer genomes have already been implemented in publicly-available software packages such as the MATLAB package SigProfiler [22], which identified APOBEC3-related mutation signatures in many different cancers [23–25]. Other techniques such as log-linear regression have been used to identify context and other factors associated with point mutations [26]. Our method determines how sequences may have evolved to either increase or decrease the number of motifs that may be targeted by enzymes such as AID and APOBEC3.

## Conclusion

We present a novel method for determining over- and /under- representation of AID/APOBEC cytidine deaminase mutation motifs. This program allows the user to choose how he/she wishes to correct for various sequence features (GC-content, dinucleotide content and codon bias) which might influence the level of over- and /under- representation. For example, if one is trying to measure over- or /under- representation and correct for codon bias, then the dn23 shuffle type may be a preferred option. In particular, it may be important to closely consider GC-content of the sequences, since this seems to be a significant factor in determining mutation motif over/under representation [15]. Both the gc3 and n3 shuffle methods correct for GC content.

## Abbreviations

APOBEC: Apolipoprotein B mRNA editing enzyme, catalytic polypeptide-like
AID: Activation induced deaminase
AAV2: Adeno-associated virus 2
CDUR: Cytidine deaminase under-representation reporter
HPV: Human papillomavirus
MM: Mutation motifs
ORF: Open reading frame
ssDNA: Single stranded DNA
