# Correction to: The cytidine deaminase under-representation reporter (CDUR) as a tool to study evolution of sequences under deaminase mutational pressure

**DOI:** 10.1186/s12859-018-2259-2

**Published:** 2018-07-04

**Authors:** Maxwell Shapiro, Stephen Meier, Thomas MacCarthy

**Affiliations:** 1 Department of Applied Mathematics and Statistics, Stony Brook University, 100 Nicolls Road, Stony Brook, NY USA; 2 Laufer Center for Physical and Quantitative Biology, Stony Brook University, 100 Nicolls Road, Stony Brook, NY USA

## Correction

Following publication of the original article [1], the authors reported that Figs. 1 and 3 were interchanged. The original article has been corrected.

**Fig. 1:**
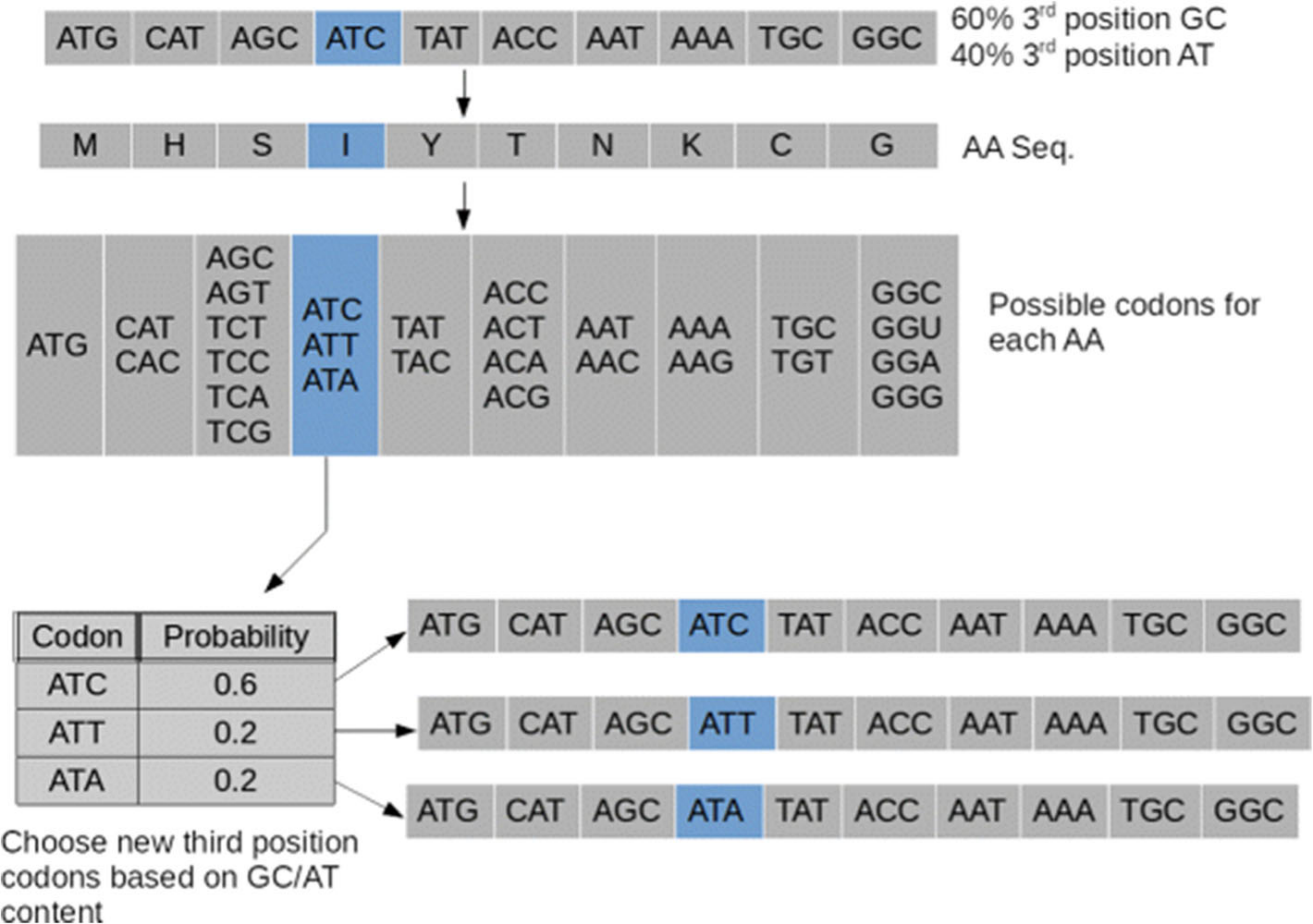
gc3 shuffle method. The choice of codons in the 4th nucleotide in the sequence (Ile) was determined by the probabilities as follows: since there is an overall GC content of 60% at the 3rd position of the codons in the subject sequence, the ATC codon will be chosen with 0.6 probability. Since the AT content is then 0.4, the other two codons ATT and ATA are chosen randomly with equal probability, conditional on the 40% AT content. Note that the shuffling occurs iteratively throughout sequence, not just one codon at a time

**Fig. 3:**
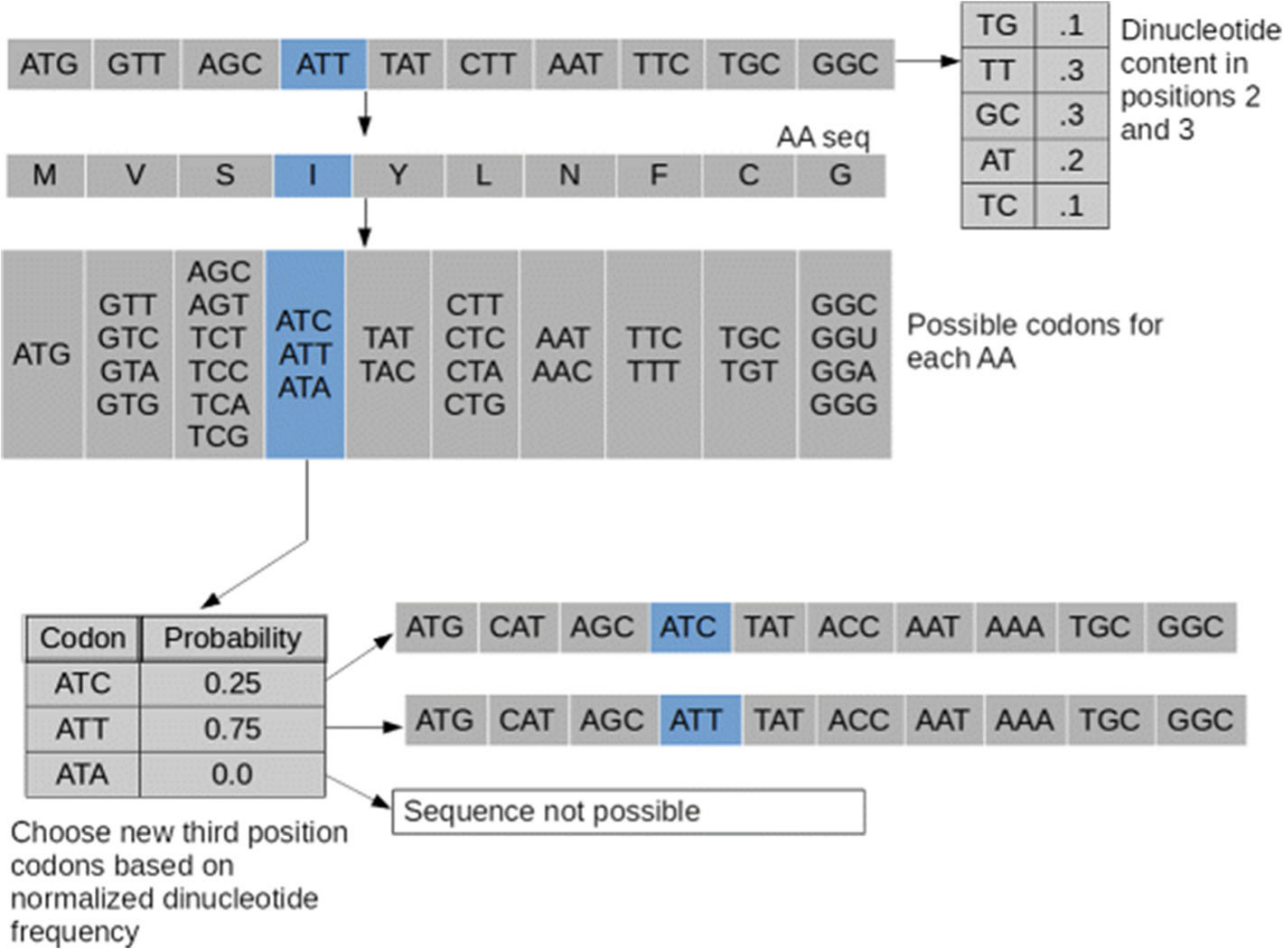
dn23 shuffle method. First the dinucleotide frequency is calculated for the 2nd and 3rd codon positions of the original sequence. Then for each amino acid, codons are chosen based on the appropriately normalized probabilities for the dinucleotides available for that amino acid

The correct versions of the figures are given below:
